# Efficacy of Single Dose of Fosfomycin Versus a Five-Day Course of Ciprofloxacin in Patients With Uncomplicated Urinary Tract Infection

**DOI:** 10.7759/cureus.24843

**Published:** 2022-05-09

**Authors:** Muhammad Mujeeb Hassan, Moeena Malik, Rabia Saleem, Amna Saleem, Khurram Zohaib, Adnan Younas Malik, Maham Javaid

**Affiliations:** 1 Department of Internal Medicine, Rawalpindi Medical University, Rawalpindi, PAK; 2 Department of Obstetrics and Gynecology, Rawalpindi Medical University, Rawalpindi, PAK; 3 Department of Pediatrics and Child Health, Islamic International Medical College (IIMCT) Pakistan Railway Hospital, Rawalpindi, PAK; 4 Department of Internal Medicine and Nephrology, Rawalpindi Medical University, Rawalpindi, PAK; 5 Department of Internal Medicine, Allama Iqbal Medical College, Lahore, PAK

**Keywords:** randomized controlled trial, multidrug-resistant bacteria, fosfomycin, uncomplicated urinary tract infection, ciprofloxacin

## Abstract

Introduction and objective: Treatment has become more challenging due to an aging population, polypharmacy and high prevalence of comorbid illness, antimicrobial antibiotic allergy or sensitivity, an increase in the number of individuals with underlying immunological or structural abnormalities, as well as the frequency of multidrug-resistant infections. Many multidrug-resistant bacteria are still susceptible to nitrofurantoin and fosfomycin, two ancient medicines. Their high urine concentrations and low toxicity give them an advantage over newer medications. This study aimed to compare the efficacy of a single dose of fosfomycin versus a five-day course of ciprofloxacin in patients with uncomplicated urinary tract infections.

Methodology and data collection procedure: This randomized control trial was conducted in the Department of Medicine, Benazir Bhutto Hospital, Rawalpindi. A total of 46 patients were enrolled. Patients were divided randomly into two groups by using the lottery method. In group A, patients were given a single 3 g dose of oral fosfomycin. In group B, patients were given oral ciprofloxacin (500 mg) daily for five days. Then patients were followed-up in the Outpatient Department (OPD) for 28 days. After 28 days, patients were evaluated for resolution of symptoms (as per operational definition). Patients in whom symptoms would not be resolved were managed as per standard protocol. All this information was recorded on proforma.

Results: The average age of the patients in group A was 39.41±9.80 years while in group B that was 41.32±17.76 years. In both groups, 23 females were equally divided. The mean duration of symptoms in group A was 4.78±1.98 days while in group B was 4.95±1.29 days. The minimum duration of symptoms was three days and the maximum was 10 days. In group A, there were 15 (65.21%) patients with efficacy achieved, and among eight (34.78%) patients, efficacy was not achieved while in group B, there were 15 (65.21%) patients in which efficacy was achieved, and among eight (34.78%) patients, efficacy was not achieved. There was no significant association between efficacy and study groups as the p-value was not significant (p=0.87).

Conclusion: The conclusion of the study was that in the treatment of simple urinary infections, a single dose of fosfomycin had equal efficacy and tolerability as a five-day course of ciprofloxacin.

## Introduction

Treatment has become more challenging due to an aging population, polypharmacy and high prevalence of comorbid illness, antimicrobial antibiotic allergy or sensitivity, an increase in the number of individuals with underlying immunological or structural abnormalities, as well as the frequency of multidrug-resistant infections [1,2].

A lower urinary tract infection (UTI) is defined as an infection of the bladder and associated structures and an uncomplicated UTI is defined as an infection caused by the typical pathogens in people with a normal urinary tract and kidney function, and no predisposing comorbidities. Uncomplicated UTIs are the common indications for antibiotic use in the community. The Gram-negative bacteria that can cause infection, on the other hand, are growing increasingly resistant to drugs [3]. Many multidrug-resistant bacteria are still susceptible to nitrofurantoin and fosfomycin, two ancient medicines. Their high urine concentrations and low toxicity give them an advantage over newer medications. Fosfomycin could be used to treat people with simple urinary tract infections caused by resistant bacteria [4].

In vitro, fosfomycin has shown promise against multidrug-resistant urinary infections; yet, clinical data is limited [5]. Oral fosfomycin is generally well tolerated and has few significant side effects. Only 5% of people report experiencing adverse effects, the most common symptom is diarrhea [6]. The most frequent bacterial infections in the population are uncomplicated UTIs. Ciprofloxacin has recently become a good treatment option. But, as ciprofloxacin resistance has grown, the efficacy of existing dosage regimens became questionable [7].

Ciprofloxacin is indeed a safe and effective treatment option for individuals suffering from acute or severe urinary tract infections [8]. One study found that the efficacy of a single dose of fosfomycin was achieved in 58% of patients [9]. While the efficacy of a five-day course of ciprofloxacin was achieved in 89% of patients [10]. But one trial found that the efficacy of single-dose fosfomycin was 96% while ciprofloxacin was 94% for uncomplicated urinary tract infection (p>0.05) [11].

The rationale of this study was to compare the efficacy of a single dose of fosfomycin versus a five-day course of ciprofloxacin in patients with uncomplicated urinary tract infections. Literature showed that ciprofloxacin is more beneficial in improving the condition of patients than fosfomycin. However, in the literature, it was discovered that there is no difference between a five-day course of ciprofloxacin and a single fosfomycin dose. So we planned to conduct this study to find if there is any difference in both drugs for the management of uncomplicated urinary tract infections in a local setting. This may help to improve our practice and in the future, on the basis of the results of this study, we will be able to implement a more beneficial drug for patients with uncomplicated urinary tract infections.

This study was undertaken under the consideration of the local antibiotic sensitivity and susceptibility and Institution's guidelines were followed for the use of these two drugs to treat uncomplicated urinary tract infections. Though the use of ciprofloxacin for uncomplicated urinary tract infections differs from the general guidelines, the institutional guidelines mandate the use of this drug due to increasing bacterial resistance to the traditional first-line antibiotics against uncomplicated urinary tract infections.

## Materials and methods

This randomized controlled trial was conducted from December 31, 2019, to July 01, 2020, in the Medicine Department, Benazir Bhutto Hospital, Rawalpindi Medical University, Rawalpindi. A sample size of 46 cases; 23 cases in each group are calculated with 80% power of the study, 95% confidence interval, and taking an expected percentage of efficacy, i.e., 58% with fosfomycin while 89% with ciprofloxacin [10].

Female patients with ages between 20 years and 70 years presenting with uncomplicated urinary tract infection were included. Patients with comorbid conditions including diabetes (blood sugar random {BSR} >200 mg/dL), hypertension (blood pressure {BP} ≥160/110 mmHg), hepatic disease (aspartate aminotransferase {AST} >40 IU, alanine aminotransferase {ALT} >40 IU), renal dysfunction (creatinine >1.2 mg/dL), and already taken the trial drug, patients with recurrent or relapse urinary tract infection were excluded from the study.

Demographic information including age, marital status, and duration of symptoms was also noted. Then patients were randomly divided into two groups by using the lottery method. In group A, patients were given a single 3 g dose of oral fosfomycin. In group B, patients were given oral ciprofloxacin (500 mg) daily for five days. Then patients were followed-up in Outpatient Department (OPD) for 28 days. After 28 days, patients were evaluated for the resolution of symptoms. Patients in whom symptoms would not be resolved were managed as per standard protocol. Data were analyzed through SPSS version 20 (Armonk, NY: IBM Corp.). Age and duration of symptoms were presented as mean and SD. Marital status and efficacy were presented as percentage and frequency. Both groups were compared for efficacy by using the chi-square test. Data were stratified for age, gender, marital status, and duration of symptoms. For each stratum, the chi-square test was used to compare efficacy in both groups. P-values less than 0.05 were considered significant.

## Results

A total of 46 patients were enrolled. In group A, the mean age was 39.41±9.80 years and in group B, the mean age was 41.32±17.76 years. The mean duration of symptoms in group A was 4.78±1.98 days and in group B was 4.95±1.29 days. A total of 23 females were divided equally into both groups (Table 1).

**Table 1:** Statistics of age and duration of symptoms.

Variables	Group A fosfomycin	Group B ciprofloxacin
Age (years), mean±SD	39.41±9.80	41.32±17.76
Duration of symptoms (days), mean±SD	4.78±1.98	4.95±1.29

In group A, there were 19 (82.6%) patients who were married and four (17.39%) were unmarried on the other side in group B, there were 22 (95.65%) married and one (4.34%) unmarried patient. In group A, there were 15 (65.21%) patients with efficacy achieved and among eight (34.78%) patients, efficacy was not achieved while in group B, there were 15 (65.21%) patients in which efficacy was achieved and among eight (34.78%) patients, efficacy was not achieved (Table 2). There was no significant association between efficacy and study groups as the p-value was not significant (p=0.87).

**Table 2:** Frequency of marital status and efficacy of treatment.

Variables		Group A fosfomycin	Group B ciprofloxacin
Marital status	Married (n)	19 (82.6%)	22 (95.65%)
	Unmarried (n)	4 (17.39%)	1 (4.34%)
Efficacy of treatment	Achieved (n)	15 (65.21%)	15 (65.21%)
	Not achieved (n)	8 (34.78%)	8 (34.78%)

No significant difference in efficacy was present between both groups in all of the age groups, e.g., 20-40 years, 41-60 years, and more than 60 years as the p-values were not significant (p=0.22, 0.085, and 0.85, respectively). Moreover, marital status and duration of symptoms also had no significant association in efficacy between both groups (Table 3).

**Table 3:** Efficacy compared in groups stratified for age groups, gender, marital status, and duration of symptoms.

Variables		Group A efficacy		Group B efficacy		p-Value
		Achieved	Not achieved	Achieved	Not achieved	
Age (years)	20-40	9 (69.23%)	4 (30.76%)	3 (42.85%)	4 (57.14%)	0.22
	41-60	4 (57.1%)	3 (42.85%)	10 (83.33%)	2 (16.66%)	0.08
	>60	2 (66.7%)	1 (33.3%)	2 (50%)	2 (50%)	0.75
Marital status	Married	11 (61.11%)	7 (36.84%)	15 (71.4%)	6 (28.6%)	0.38
	Unmarried	4 (80%)	1 (20%)	0	2 (100%)	0.54
Duration of symptoms (days)	2-5	13 (76.4%)	4 (23.52%)	11 (73.33%)	4 (26.66%)	0.95
	6-10	2 (33.33%)	4 (66.6%)	4 (50.0%)	4 (50.0%)	0.52

## Discussion

Urinary tract infections (UTIs) are the most prevalent infections in humans [12,13]. It’s estimated that half of all women will have at least one UTI in their lifetime, with a quarter of those suffering from recurring infections. According to Maladkar and Revandkar, the cure rates for fosfomycin and ciprofloxacin treatment did not differ significantly (83.0-80.0%) in their study. Similar are the findings of our study as in our study there was no significant association between study groups and efficacy [14].

Ciprofloxacin resistance in *Escherichia coli* has been reported to be as high as 58-63% in recent studies [15]. Ciprofloxacin use only within the previous six months or one year has been found to be such a strong separating factor in view of resistance pattern in Turkish studies [16]. Fluoroquinolones are the most commonly given medicine in the empirical treatment of renal infections, which are prescribed for many 77.0% of cases [17]. In light of these findings, frequent empirical fluoroquinolone medicine is assumed to be the leading source of resistance in *E. coli*. Antibiotics under availability, as well as the affordability of ciprofloxacin therapy, are the additional factors contributing to the rate of use & increases in resistance.

Despite the complex nature of the patients and diseases described, Derington et al. presented their findings. Fosfomycin was associated with a clinical response rate of 66.0% which is almost similar to the findings of our study as in our study this success rate with fosfomycin was 63.3% [18]. The study by Derington et al. found that patients treated with Fosfomycin were most often older patients with at least one previous UTI, several comorbidities, and a history of repeated antibiotic exposure within the previous 90 days [18]. *E. coli*, Klebsiella, Enterococcus, and Pseudomonas spp. were the most common infections treated with fosfomycin; these bacteria were linked to increased risk of non-susceptibility to antibiotics and multidrug resistance.

The microbiological effectiveness of the two antibiotics appears to be considerably varied in the study. But, the outcomes were similar once evaluated in terms of improvement in patients’ findings and bacterial eradication. In this case, factors like antibiotic efficacy in vivo and the concentration of tissue are important. Fosfomycin had two distinguishing characteristics. Clinical remission and bacteriological elimination rates were reduced and the period of remission of symptoms was prolonged, despite its excellent microbiological sensitivity. It’s supposed that although fosfomycin was stated to reach suitable concentrations in the urine; It's in vivo effectiveness and the urine concentration can be reduced.

In another study, the bacteriological cure rates for fosfomycin and ciprofloxacin, which did not reach a statistically significant difference (p=0.05) and were similar are the findings of our study as in our study the cure rate for fosfomycin and ciprofloxacin. The high diffusion capacity is assumed to be responsible for this result, ciprofloxacin concentrations in the urine are greater, as well as medicine dosage (twice a day 500 mg for five days) [19].

People with UTIs caused by (or carrying) a multi-resistant Enterobacteriaceae are more likely to require complete intravenous antibiotic regimens, raising the risk for complications, psychological load, and larger costs of health care, partially owing to the upcoming extended period of hospital duration [20]. FT could be a good option for focused stepdown treatment of *E. coli* UTI. In observational studies involving UTIs, FT was found to be non-inferior to carbapenems [21]. Lower bacteriologic responses have been found after a one-dose of fosfomycin treatment for uncomplicated UTIs [22]. Clinical and microbiologic success rates of 78.0-95.0% and 62-98% were reported in previous studies with multiple-dose fosfomycin (MDF) treatment (range: 2.0-6.0 doses) for out-patient treatment of uncomplicated UTI.

There may be some possible limitations to this study. First of all, there were no previous local trial studies on this topic. So, it was really hard to get accurate data on local culture and sensitivities on this topic. This is an important knowledge gap and represents an opportunity for further development in this area of the field. The second minor limitation was the lack of proper funding for this project, which resulted in the use of available resources and not requesting the need to run culture and sensitivities in the treatment of the patients, although the choice of the antibiotics was according to the institutional guidelines which were developed considering the usual pathogens causing the urinary tract infections in the local population. By getting the approval of proper funding in the future by collaboration with the research institutes, we aim to minimize the urge from deviating away from the standard clinical practice.

## Conclusions

Urinary tract infections (UTIs) are the most prevalent infections in women and by one of the estimates about half of all women will have at least one UTI in their lifetime, with many of those would go on to have the recurrent infections. In the treatment of simple urinary infections, a single dose of fosfomycin had equal efficacy and tolerability as a five-day course of ciprofloxacin. Despite being only a single dose course and having better compliance than the ciprofloxacin, the results of fosfomycin treatment were non-inferior to ciprofloxacin treatment.
